# 3-(4-Fluoro­phenyl)-1-[1′-(4-fluoro­phenyl)-2-oxo-5′,6′,7′,7a′-tetra­hydro-1*H*-indole-3(2*H*)-spiro-3′(2′*H*)-1*H*′-pyrrol­izin-2′-yl]prop-2-en-1-one

**DOI:** 10.1107/S1600536808025774

**Published:** 2008-08-16

**Authors:** S. Nirmala, R. Murugan, E. Theboral Sugi Kamala, L. Sudha, S. Sriman Narayanan

**Affiliations:** a Department of Physics, Easwari Engineering College, Ramapuram, Chennai 600 089, India; b Department of Analytical Chemistry, University of Madras, Guindy Campus, Chennai 600 025, India; c Department of Physics, SRM University, Ramapuram Campus, Chennai 600 089, India

## Abstract

In the title compound, C_29_H_24_F_2_N_2_O_2_, one of the pyrrolidine rings of the pyrrolizine system is disordered over two sites, with occupancy factors 0.734:0.266 (12). Both components of the disordered pyrrolidine ring adopt envelope conformations, whereas the other pyrrolidine ring adopts a twist conformation. The mol­ecules are linked into centrosymmetric dimers by N—H⋯O hydrogen bonds and the dimers are connected *via* C—H⋯π inter­actions. The crystal structure is also stabilized by inter­molecular C—H⋯F hydrogen bonds.

## Related literature

For related literature, see: Atal (1978 ▶); Cordel (1981 ▶); Cremer & Pople (1975 ▶); Denny (2001 ▶); Harris & Uhle (1960 ▶); Ho *et al.* (1986 ▶); Rajeswaran *et al.* (1999 ▶); Ramesh *et al.* (2007 ▶); Stevenson *et al.* (2000 ▶).
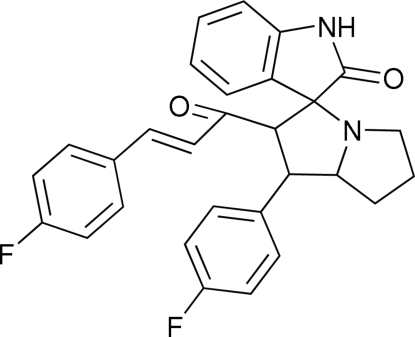


## Experimental

### 

#### Crystal data

C_29_H_24_F_2_N_2_O_2_
*M* *_r_* = 470.50
Triclinic, 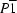

*a* = 8.3985 (4) Å
*b* = 12.0018 (6) Å
*c* = 12.5628 (6) Å
α = 96.464 (2)°
β = 104.348 (2)°
γ = 104.144 (2)°
*V* = 1169.23 (10) Å^3^
*Z* = 2
Mo *K*α radiation
μ = 0.10 mm^−1^
*T* = 293 (2) K
0.30 × 0.20 × 0.20 mm

#### Data collection

Bruker KappaAPEXII diffractometer
Absorption correction: multi-scan (Blessing, 1995 ▶) *T*_min_ = 0.972, *T*_max_ = 0.981
25777 measured reflections
6118 independent reflections
4000 reflections with *I* > 2σ(*I*)
*R*_int_ = 0.032

#### Refinement

*R*[*F* ^2^ > 2σ(*F* ^2^)] = 0.049
*wR*(*F* ^2^) = 0.168
*S* = 1.05
6118 reflections
326 parameters
H-atom parameters constrained
Δρ_max_ = 0.27 e Å^−3^
Δρ_min_ = −0.24 e Å^−3^

### 

Data collection: *APEX2* (Bruker, 2004 ▶); cell refinement: *APEX2* and *SAINT* (Bruker, 2004 ▶); data reduction: *SAINT* and *XPREP* (Bruker, 2004 ▶); program(s) used to solve structure: *SHELXS97* (Sheldrick, 2008 ▶); program(s) used to refine structure: *SHELXL97* (Sheldrick, 2008 ▶); molecular graphics: *ORTEP-3* (Farrugia, 1997 ▶); software used to prepare material for publication: *PLATON* (Spek, 2003 ▶).

## Supplementary Material

Crystal structure: contains datablocks I, global. DOI: 10.1107/S1600536808025774/bt2765sup1.cif

Structure factors: contains datablocks I. DOI: 10.1107/S1600536808025774/bt2765Isup2.hkl

Additional supplementary materials:  crystallographic information; 3D view; checkCIF report

## Figures and Tables

**Table 1:** Hydrogen-bond geometry (Å, °) *Cg*1 is the centroid of the ring composed of atoms C8–C13.

*D*—H⋯*A*	*D*—H	H⋯*A*	*D*⋯*A*	*D*—H⋯*A*
N2—H2⋯O2^i^	0.86	2.09	2.922 (2)	164
C9—H9⋯F2^ii^	0.93	2.55	3.165 (3)	124
C28—H28⋯*Cg*1^iii^	0.93	2.97	3.886 (3)	169

Symmetry codes: (i) 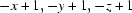; (ii) 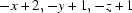; (iii) 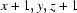.

**Fig. 1.**
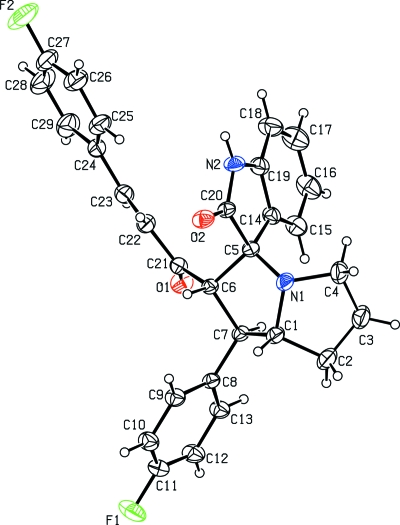
The molecular structure of the title compound with 30% probability displacement ellipsoids.

**Fig. 2.**
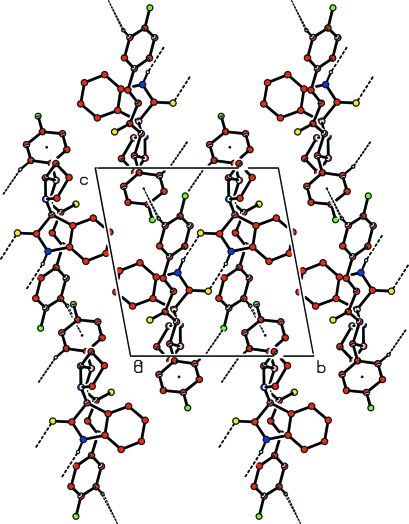
The packing of the molecules viewed down the a axis. Dashed lines indicate hydrogen bonds. H atoms not involed in hydrogen bonds have been omitted.

## supplementary crystallographic information

### Comment

The spiro ring system is a frequently encountered structural motif in many pharmacologically relevant alkaloids (Cordel, 1981). Pyrrolizidine alkaloids occur in more than 40 genera, and are responsible for heavy losses of livestock and poisoning in man due to their hepatotoxity. These alkaloids are also reported to possess a number of other biological activities (Atal, 1978) and are used as DNA minor groove alkylating agents (Denny, 2001). Indole compounds can be used as bioactive drugs (Stevenson *et al.*, 2000). Indole derivatives exhibit anti-allergic, central nervous system depressant and muscle relaxant properties (Harris & Uhle, 1960; Ho *et al.*, 1986). Indoles have also been proved to display high aldose reductase inhibitory activity (Rajeswaran *et al.*, 1999). In view of this biological importance, an X-ray study of the title compound, (I), was carried out.

An *ORTEP* (Farrugia, 1997) plot of the molecule is shown in Fig. 1. The pyrrolizine ring system is folded about the bridging N1—C1 bond, as observed in related structures (Ramesh *et al.*, 2007). The sum of angles at N1 (339°) is in accordance with *sp*^3^ hybridization. The fluorine atoms attached at C11 and C27 are coplanar with the phenyl rings C8–C13 and C24–C29 as indicated by the torsion angles F1—C11—C12—C13 [179.4 (2)°] and F2—C27—C28—C29 [178.6 (2)°] respectively. The indole moiety is planar [maximum deviation of -0.045 (3)° from the least square plane defined by all non hydrogen atoms in the molecule] and makes a dihedral angle of 37 (6)° with the ring C8–C13, 53.7 (6)° with the ring C24–C29. Both the phenyl rings are nearly perpendicular to each other and are oriented at an angle of 82.1 (7)° with respect to each other. In the pyrrolizine ring system, the pyrrolidine ring (N1/C1/C5/C6/C7) adopts a twist conformation with Cremer & Pople (1975) puckering parameters q_2_ and φ of 0.425 (2) Å and 123.6 (3)° respectively. The disordered pyrrolidine ring adopts an envelope conformation in both the major and minor conformers with Cremer & Pople (1975) puckering parameters q_2_ and φ of 0.323 (4) Å and -59.2 (8)°, respectively, for the major conformer (N1/C1–C4), and 0.271 (13) Å and 96.8 (1)°, respectively, for the minor conformer (N1/C1/C2/C3A/C4). Atom C3/C3A deviates by -0.484 (3) Å / 0.402 (9) Å from the least square planes through the remaining four atoms N1, C1, C2 and C4.

In the crystal structure, symmetry-related molecules form N—H···O hydrogen-bonded dimers and the dimers are linked *via *C—H···π interactions involving C8–C13 benzene rings. The crystal structure is also stabilized by intermolecular C—H···F hydrogen bonds (Table 1) and van der Waals forces.

### Experimental

A solution of (1*E*,6*E*)-4-(4-flurobenzylidene)-1,7-bis(4- flurophenyl)hepta-1,6-diene-3,5-dione (1 mmol), isatin (1 mmol) and *L*-proline (1 mmol) in aqueous methanol (20 ml) was refluxed until the disappearance of starting material as evidenced by TLC.  The solvent was removed under reduced pressure and the crude product was purified by column chromatography using petroleum ether–ethylacetate (5:1) as eluent. The final product was recrystallized in ethanol and chloroform (2:8).

### Refinement

The C atom of pyrrolizine unit is disordered over two positions with site occupancies of C3 = 0.734 (1) and C3A = 0.266 (1). H atoms were placed in idealized positions and allowed to ride on their parent atoms, with C—H = 0.93, 0.98, and 0.97 Å for aromatic, methine and methylene respectively, and with *U*_iso_(H) = 1.2Ueq(C) for all H atoms.

### Crystal data

**Table:**

C_29_H_24_F_2_N_2_O_2_	*Z* = 2
*M**_r_* = 470.50	*F*_000_ = 492
Triclinic, *P*1	*D*_x_ = 1.336 Mg m^−^^3^
Hall symbol: -P 1	Mo *K*α radiation λ = 0.71073 Å
*a* = 8.3985 (4) Å	Cell parameters from 7237 reflections
*b* = 12.0018 (6) Å	θ = 2.2–27.9º
*c* = 12.5628 (6) Å	µ = 0.10 mm^−^^1^
α = 96.464 (2)º	*T* = 293 (2) K
β = 104.348 (2)º	Prism, colourless
γ = 104.144 (2)º	0.30 × 0.20 × 0.20 mm
*V* = 1169.23 (10) Å^3^	

### Data collection

**Table:**

Bruker KappaAPEXII diffractometer	6118 independent reflections
Radiation source: fine-focus sealed tube	4000 reflections with *I* > 2σ(*I*)
Monochromator: graphite	*R*_int_ = 0.032
*T* = 293(2) K	θ_max_ = 29.0º
ω and φ scans	θ_min_ = 1.7º
Absorption correction: multi-scan(Blessing, 1995)	*h* = −10→11
*T*_min_ = 0.972, *T*_max_ = 0.981	*k* = −16→16
25777 measured reflections	*l* = −17→17

### Refinement

**Table:**

Refinement on *F*^2^	Secondary atom site location: difference Fourier map
Least-squares matrix: full	Hydrogen site location: inferred from neighbouring sites
*R*[*F*^2^ > 2σ(*F*^2^)] = 0.049	H-atom parameters constrained
*wR*(*F*^2^) = 0.168	*w* = 1/[σ^2^(*F*_o_^2^) + (0.0744*P*)^2^ + 0.4073*P*] where *P* = (*F*_o_^2^ + 2*F*_c_^2^)/3
*S* = 1.05	(Δ/σ)_max_ < 0.001
6118 reflections	Δρ_max_ = 0.27 e Å^−^^3^
326 parameters	Δρ_min_ = −0.24 e Å^−^^3^
Primary atom site location: structure-invariant direct methods	Extinction correction: none

### Special details

**Table:**

Geometry. All e.s.d.'s (except the e.s.d. in the dihedral angle between two l.s. planes) are estimated using the full covariance matrix. The cell e.s.d.'s are taken into account individually in the estimation of e.s.d.'s in distances, angles and torsion angles; correlations between e.s.d.'s in cell parameters are only used when they are defined by crystal symmetry. An approximate (isotropic) treatment of cell e.s.d.'s is used for estimating e.s.d.'s involving l.s. planes.
Refinement. Refinement of *F*^2^ against ALL reflections. The weighted *R*-factor *wR* and goodness of fit *S* are based on *F*^2^, conventional *R*-factors *R* are based on *F*, with *F* set to zero for negative *F*^2^. The threshold expression of *F*^2^ > σ(*F*^2^) is used only for calculating *R*-factors(gt) *etc*. and is not relevant to the choice of reflections for refinement. *R*-factors based on *F*^2^ are statistically about twice as large as those based on *F*, and *R*- factors based on ALL data will be even larger.

### Fractional atomic coordinates and isotropic or equivalent isotropic displacement parameters (Å^2^)

**Table:**

	*x*	*y*	*z*	*U*_iso_*/*U*_eq_	Occ. (<1)
C1	0.2007 (2)	0.28284 (17)	0.05257 (14)	0.0421 (4)	
H1	0.2339	0.3580	0.0288	0.051*	
C2	0.0256 (3)	0.2104 (2)	−0.02359 (18)	0.0643 (6)	
H2A	−0.0359	0.2602	−0.0613	0.077*	0.734 (12)
H2B	0.0378	0.1529	−0.0795	0.077*	0.734 (12)
H2C	0.0235	0.1297	−0.0446	0.077*	0.266 (12)
H2D	−0.0067	0.2425	−0.0906	0.077*	0.266 (12)
C3	−0.0654 (5)	0.1528 (5)	0.0501 (3)	0.0599 (12)	0.734 (12)
H3A	−0.0397	0.0795	0.0603	0.072*	0.734 (12)
H3B	−0.1883	0.1377	0.0199	0.072*	0.734 (12)
C3A	−0.0922 (11)	0.2214 (18)	0.0519 (8)	0.066 (4)	0.266 (12)
H3C	−0.1405	0.2858	0.0381	0.079*	0.266 (12)
H3D	−0.1857	0.1502	0.0353	0.079*	0.266 (12)
C4	0.0019 (2)	0.2405 (2)	0.16055 (19)	0.0590 (6)	
H4A	−0.0671	0.2945	0.1615	0.071*	0.734 (12)
H4B	0.0003	0.2004	0.2233	0.071*	0.734 (12)
H4C	−0.0017	0.1669	0.1861	0.071*	0.266 (12)
H4D	−0.0426	0.2873	0.2075	0.071*	0.266 (12)
C5	0.3222 (2)	0.28514 (14)	0.24864 (13)	0.0360 (4)	
C6	0.4579 (2)	0.28659 (14)	0.18298 (13)	0.0331 (3)	
H6	0.5117	0.3681	0.1806	0.040*	
C7	0.3473 (2)	0.22630 (14)	0.06553 (13)	0.0350 (3)	
H7	0.3011	0.1432	0.0665	0.042*	
C8	0.4353 (2)	0.23548 (14)	−0.02503 (13)	0.0350 (3)	
C9	0.5264 (2)	0.34286 (15)	−0.04026 (15)	0.0432 (4)	
H9	0.5345	0.4105	0.0074	0.052*	
C10	0.6052 (3)	0.35163 (17)	−0.12456 (16)	0.0494 (5)	
H10	0.6660	0.4238	−0.1343	0.059*	
C11	0.5911 (3)	0.25112 (18)	−0.19290 (16)	0.0489 (5)	
C12	0.5029 (3)	0.14399 (17)	−0.18227 (16)	0.0510 (5)	
H12	0.4951	0.0772	−0.2309	0.061*	
C13	0.4251 (2)	0.13654 (16)	−0.09742 (15)	0.0437 (4)	
H13	0.3648	0.0637	−0.0888	0.052*	
C14	0.2918 (2)	0.18120 (15)	0.30613 (15)	0.0403 (4)	
C15	0.2356 (3)	0.06244 (17)	0.26645 (18)	0.0527 (5)	
H15	0.2022	0.0338	0.1900	0.063*	
C16	0.2291 (3)	−0.01396 (19)	0.3409 (2)	0.0677 (7)	
H16	0.1887	−0.0942	0.3143	0.081*	
C17	0.2819 (4)	0.0282 (2)	0.4534 (2)	0.0740 (7)	
H17	0.2794	−0.0243	0.5024	0.089*	
C18	0.3391 (3)	0.1468 (2)	0.49613 (19)	0.0628 (6)	
H18	0.3744	0.1751	0.5727	0.075*	
C19	0.3414 (2)	0.22114 (16)	0.42044 (15)	0.0445 (4)	
C20	0.3922 (2)	0.38838 (15)	0.34997 (13)	0.0371 (4)	
C21	0.5974 (2)	0.23417 (15)	0.23597 (14)	0.0381 (4)	
C22	0.7119 (2)	0.30084 (16)	0.34466 (15)	0.0430 (4)	
H22	0.7106	0.3774	0.3656	0.052*	
C23	0.8167 (2)	0.25794 (18)	0.41428 (16)	0.0459 (4)	
H23	0.8278	0.1853	0.3884	0.055*	
C24	0.9164 (2)	0.31492 (18)	0.52817 (16)	0.0464 (4)	
C25	0.8823 (3)	0.4085 (2)	0.58393 (18)	0.0585 (5)	
H25	0.7964	0.4383	0.5466	0.070*	
C26	0.9719 (3)	0.4583 (2)	0.6928 (2)	0.0680 (6)	
H26	0.9466	0.5201	0.7303	0.082*	
C27	1.0995 (3)	0.4140 (2)	0.7441 (2)	0.0720 (7)	
C28	1.1373 (4)	0.3227 (3)	0.6940 (2)	0.0853 (9)	
H28	1.2246	0.2944	0.7319	0.102*	
C29	1.0442 (3)	0.2719 (2)	0.5854 (2)	0.0700 (7)	
H29	1.0677	0.2079	0.5502	0.084*	
N1	0.17837 (18)	0.30269 (14)	0.16568 (12)	0.0426 (4)	
N2	0.3950 (2)	0.34354 (13)	0.44352 (12)	0.0453 (4)	
H2	0.4259	0.3850	0.5095	0.054*	
O1	0.60955 (18)	0.14274 (12)	0.19211 (12)	0.0536 (4)	
O2	0.44108 (18)	0.49119 (11)	0.34531 (10)	0.0478 (3)	
F2	1.1884 (3)	0.46239 (17)	0.85163 (14)	0.1156 (7)	
F1	0.6690 (2)	0.25907 (13)	−0.27532 (12)	0.0781 (4)	

### Atomic displacement parameters (Å^2^)

**Table:**

	*U*^11^	*U*^22^	*U*^33^	*U*^12^	*U*^13^	*U*^23^
C1	0.0388 (9)	0.0485 (10)	0.0329 (9)	0.0100 (7)	0.0052 (7)	−0.0006 (7)
C2	0.0424 (10)	0.0880 (17)	0.0456 (11)	0.0135 (10)	−0.0019 (9)	−0.0116 (11)
C3	0.0409 (16)	0.062 (3)	0.0602 (19)	0.0008 (15)	0.0059 (13)	−0.0076 (17)
C3A	0.036 (4)	0.094 (11)	0.055 (5)	0.011 (5)	0.001 (3)	0.003 (6)
C4	0.0372 (9)	0.0743 (15)	0.0586 (13)	0.0093 (9)	0.0146 (9)	−0.0037 (11)
C5	0.0377 (8)	0.0356 (8)	0.0290 (8)	0.0045 (6)	0.0097 (6)	−0.0038 (6)
C6	0.0364 (8)	0.0315 (8)	0.0271 (7)	0.0046 (6)	0.0082 (6)	0.0009 (6)
C7	0.0391 (8)	0.0324 (8)	0.0270 (8)	0.0040 (6)	0.0071 (6)	−0.0015 (6)
C8	0.0381 (8)	0.0365 (8)	0.0271 (7)	0.0090 (6)	0.0064 (6)	0.0026 (6)
C9	0.0528 (10)	0.0344 (9)	0.0389 (9)	0.0082 (7)	0.0132 (8)	0.0016 (7)
C10	0.0574 (11)	0.0436 (10)	0.0467 (11)	0.0069 (8)	0.0192 (9)	0.0125 (8)
C11	0.0559 (11)	0.0577 (12)	0.0377 (10)	0.0160 (9)	0.0210 (8)	0.0101 (8)
C12	0.0675 (12)	0.0449 (10)	0.0417 (10)	0.0146 (9)	0.0232 (9)	−0.0022 (8)
C13	0.0555 (10)	0.0345 (9)	0.0377 (9)	0.0059 (7)	0.0171 (8)	0.0005 (7)
C14	0.0434 (9)	0.0374 (9)	0.0388 (9)	0.0056 (7)	0.0173 (7)	0.0021 (7)
C15	0.0644 (12)	0.0398 (10)	0.0519 (11)	0.0049 (9)	0.0259 (10)	0.0012 (8)
C16	0.0881 (17)	0.0403 (11)	0.0809 (17)	0.0104 (11)	0.0425 (14)	0.0127 (11)
C17	0.0954 (19)	0.0603 (15)	0.0785 (18)	0.0231 (13)	0.0365 (15)	0.0334 (13)
C18	0.0795 (15)	0.0646 (14)	0.0476 (12)	0.0178 (12)	0.0228 (11)	0.0194 (10)
C19	0.0482 (10)	0.0450 (10)	0.0386 (9)	0.0084 (8)	0.0155 (8)	0.0049 (8)
C20	0.0393 (8)	0.0384 (9)	0.0291 (8)	0.0086 (7)	0.0080 (6)	−0.0027 (7)
C21	0.0392 (8)	0.0396 (9)	0.0358 (9)	0.0092 (7)	0.0128 (7)	0.0076 (7)
C22	0.0414 (9)	0.0442 (10)	0.0404 (9)	0.0111 (7)	0.0087 (7)	0.0052 (8)
C23	0.0439 (9)	0.0536 (11)	0.0443 (10)	0.0161 (8)	0.0156 (8)	0.0132 (8)
C24	0.0389 (9)	0.0558 (11)	0.0418 (10)	0.0086 (8)	0.0089 (8)	0.0151 (8)
C25	0.0564 (12)	0.0670 (14)	0.0446 (11)	0.0175 (10)	0.0003 (9)	0.0117 (10)
C26	0.0739 (15)	0.0629 (14)	0.0493 (12)	0.0054 (12)	0.0016 (11)	0.0049 (11)
C27	0.0622 (14)	0.0758 (17)	0.0485 (13)	−0.0085 (12)	−0.0116 (11)	0.0164 (12)
C28	0.0637 (15)	0.107 (2)	0.0712 (17)	0.0280 (15)	−0.0137 (13)	0.0259 (16)
C29	0.0569 (13)	0.0871 (17)	0.0663 (15)	0.0310 (12)	0.0057 (11)	0.0184 (13)
N1	0.0354 (7)	0.0516 (9)	0.0343 (7)	0.0099 (6)	0.0066 (6)	−0.0052 (6)
N2	0.0584 (9)	0.0433 (9)	0.0272 (7)	0.0071 (7)	0.0114 (7)	−0.0028 (6)
O1	0.0599 (8)	0.0498 (8)	0.0526 (8)	0.0225 (7)	0.0145 (7)	0.0033 (6)
O2	0.0624 (8)	0.0360 (7)	0.0370 (7)	0.0084 (6)	0.0096 (6)	−0.0023 (5)
F2	0.1142 (14)	0.1158 (14)	0.0608 (10)	−0.0031 (11)	−0.0354 (10)	0.0071 (9)
F1	0.1005 (11)	0.0816 (10)	0.0676 (9)	0.0190 (8)	0.0574 (8)	0.0156 (7)

### Geometric parameters (Å, °)

**Table:**

C1—N1	1.479 (2)	C11—F1	1.356 (2)
C1—C2	1.524 (3)	C11—C12	1.357 (3)
C1—C7	1.529 (2)	C12—C13	1.382 (3)
C1—H1	0.9800	C12—H12	0.9300
C2—C3	1.468 (4)	C13—H13	0.9300
C2—C3A	1.549 (11)	C14—C15	1.377 (3)
C2—H2A	0.9700	C14—C19	1.384 (2)
C2—H2B	0.9700	C15—C16	1.383 (3)
C2—H2C	0.9700	C15—H15	0.9300
C2—H2D	0.9700	C16—C17	1.367 (4)
C3—C4	1.531 (4)	C16—H16	0.9300
C3—H3A	0.9700	C17—C18	1.383 (4)
C3—H3B	0.9700	C17—H17	0.9300
C3A—C4	1.359 (9)	C18—C19	1.375 (3)
C3A—H3C	0.9700	C18—H18	0.9300
C3A—H3D	0.9700	C19—N2	1.400 (2)
C4—N1	1.469 (2)	C20—O2	1.214 (2)
C4—H4A	0.9700	C20—N2	1.344 (2)
C4—H4B	0.9700	C21—O1	1.210 (2)
C4—H4C	0.9700	C21—C22	1.469 (2)
C4—H4D	0.9700	C22—C23	1.321 (3)
C5—N1	1.463 (2)	C22—H22	0.9300
C5—C14	1.510 (2)	C23—C24	1.457 (3)
C5—C20	1.555 (2)	C23—H23	0.9300
C5—C6	1.562 (2)	C24—C29	1.381 (3)
C6—C21	1.510 (2)	C24—C25	1.383 (3)
C6—C7	1.521 (2)	C25—C26	1.372 (3)
C6—H6	0.9800	C25—H25	0.9300
C7—C8	1.503 (2)	C26—C27	1.364 (4)
C7—H7	0.9800	C26—H26	0.9300
C8—C13	1.383 (2)	C27—C28	1.346 (4)
C8—C9	1.389 (2)	C27—F2	1.353 (3)
C9—C10	1.382 (3)	C28—C29	1.376 (4)
C9—H9	0.9300	C28—H28	0.9300
C10—C11	1.361 (3)	C29—H29	0.9300
C10—H10	0.9300	N2—H2	0.8600
			
N1—C1—C2	105.62 (15)	C1—C7—H7	108.2
N1—C1—C7	105.01 (14)	C13—C8—C9	117.88 (16)
C2—C1—C7	117.17 (16)	C13—C8—C7	120.64 (15)
N1—C1—H1	109.6	C9—C8—C7	121.47 (15)
C2—C1—H1	109.6	C10—C9—C8	121.56 (17)
C7—C1—H1	109.6	C10—C9—H9	119.2
C3—C2—C1	105.6 (2)	C8—C9—H9	119.2
C1—C2—C3A	101.9 (4)	C11—C10—C9	117.82 (17)
C3—C2—H2A	110.6	C11—C10—H10	121.1
C1—C2—H2A	110.6	C9—C10—H10	121.1
C3A—C2—H2A	80.4	F1—C11—C12	118.78 (18)
C3—C2—H2B	110.6	F1—C11—C10	118.11 (18)
C1—C2—H2B	110.6	C12—C11—C10	123.12 (17)
C3A—C2—H2B	139.4	C11—C12—C13	118.41 (17)
H2A—C2—H2B	108.8	C11—C12—H12	120.8
C3—C2—H2C	78.3	C13—C12—H12	120.8
C1—C2—H2C	111.4	C12—C13—C8	121.21 (17)
C3A—C2—H2C	111.4	C12—C13—H13	119.4
H2A—C2—H2C	132.4	C8—C13—H13	119.4
C3—C2—H2D	135.4	C15—C14—C19	118.80 (18)
C1—C2—H2D	111.4	C15—C14—C5	132.45 (17)
C3A—C2—H2D	111.4	C19—C14—C5	108.66 (15)
H2B—C2—H2D	78.9	C14—C15—C16	119.7 (2)
H2C—C2—H2D	109.2	C14—C15—H15	120.2
C2—C3—C4	104.1 (3)	C16—C15—H15	120.2
C4—C3—H2C	130.6	C17—C16—C15	120.1 (2)
C2—C3—H3A	110.9	C17—C16—H16	119.9
C4—C3—H3A	110.9	C15—C16—H16	119.9
H2C—C3—H3A	76.6	C16—C17—C18	121.8 (2)
C2—C3—H3B	110.9	C16—C17—H17	119.1
C4—C3—H3B	110.9	C18—C17—H17	119.1
H2C—C3—H3B	111.9	C19—C18—C17	117.1 (2)
H3A—C3—H3B	108.9	C19—C18—H18	121.5
C4—C3A—C2	108.7 (7)	C17—C18—H18	121.5
C4—C3A—H3C	109.9	C18—C19—C14	122.56 (19)
C2—C3A—H3C	109.9	C18—C19—N2	127.44 (18)
C4—C3A—H3D	109.9	C14—C19—N2	109.98 (16)
C2—C3A—H3D	109.9	O2—C20—N2	126.12 (15)
H3C—C3A—H3D	108.3	O2—C20—C5	125.79 (15)
C3A—C4—N1	106.7 (5)	N2—C20—C5	108.05 (14)
N1—C4—C3	105.11 (19)	O1—C21—C22	123.42 (17)
C3A—C4—H4A	77.2	O1—C21—C6	121.68 (16)
N1—C4—H4A	110.7	C22—C21—C6	114.88 (15)
C3—C4—H4A	110.7	C23—C22—C21	123.53 (18)
C3A—C4—H4B	136.2	C23—C22—H22	118.2
N1—C4—H4B	110.7	C21—C22—H22	118.2
C3—C4—H4B	110.7	C22—C23—C24	125.45 (19)
H4A—C4—H4B	108.8	C22—C23—H23	117.3
C3A—C4—H4C	110.4	C24—C23—H23	117.3
N1—C4—H4C	110.4	C29—C24—C25	118.1 (2)
C3—C4—H4C	78.1	C29—C24—C23	119.8 (2)
H4A—C4—H4C	133.5	C25—C24—C23	122.09 (18)
C3A—C4—H4D	110.4	C26—C25—C24	121.7 (2)
N1—C4—H4D	110.4	C26—C25—H25	119.2
C3—C4—H4D	138.0	C24—C25—H25	119.2
H4B—C4—H4D	76.8	C27—C26—C25	117.6 (3)
H4C—C4—H4D	108.6	C27—C26—H26	121.2
N1—C5—C14	119.15 (14)	C25—C26—H26	121.2
N1—C5—C20	110.39 (14)	C28—C27—F2	118.8 (3)
C14—C5—C20	101.45 (13)	C28—C27—C26	123.1 (2)
N1—C5—C6	102.58 (13)	F2—C27—C26	118.0 (3)
C14—C5—C6	113.29 (14)	C27—C28—C29	118.7 (2)
C20—C5—C6	110.04 (13)	C27—C28—H28	120.6
C21—C6—C7	116.33 (14)	C29—C28—H28	120.6
C21—C6—C5	113.42 (13)	C28—C29—C24	120.8 (3)
C7—C6—C5	102.51 (12)	C28—C29—H29	119.6
C21—C6—H6	108.1	C24—C29—H29	119.6
C7—C6—H6	108.1	C5—N1—C4	119.79 (16)
C5—C6—H6	108.1	C5—N1—C1	110.37 (13)
C8—C7—C6	116.02 (13)	C4—N1—C1	108.80 (14)
C8—C7—C1	114.92 (14)	C20—N2—C19	111.77 (14)
C6—C7—C1	100.91 (13)	C20—N2—H2	124.1
C8—C7—H7	108.2	C19—N2—H2	124.1
C6—C7—H7	108.2		
			
N1—C1—C2—C3	−22.5 (3)	C16—C17—C18—C19	−0.3 (4)
C7—C1—C2—C3	94.0 (3)	C17—C18—C19—C14	−1.0 (3)
N1—C1—C2—C3A	13.1 (8)	C17—C18—C19—N2	−179.4 (2)
C7—C1—C2—C3A	129.6 (8)	C15—C14—C19—C18	1.0 (3)
C1—C2—C3—C4	32.4 (4)	C5—C14—C19—C18	−175.96 (19)
C3A—C2—C3—C4	−56.1 (6)	C15—C14—C19—N2	179.63 (17)
C3—C2—C3A—C4	73.3 (11)	C5—C14—C19—N2	2.7 (2)
C1—C2—C3A—C4	−26.9 (14)	N1—C5—C20—O2	−55.5 (2)
C2—C3A—C4—N1	29.4 (14)	C14—C5—C20—O2	177.23 (17)
C2—C3A—C4—C3	−63.4 (10)	C6—C5—C20—O2	57.0 (2)
C2—C3—C4—C3A	67.1 (8)	N1—C5—C20—N2	126.72 (15)
C2—C3—C4—N1	−30.7 (4)	C14—C5—C20—N2	−0.55 (17)
N1—C5—C6—C21	−162.29 (13)	C6—C5—C20—N2	−120.77 (15)
C14—C5—C6—C21	−32.54 (19)	C7—C6—C21—O1	−7.2 (2)
C20—C5—C6—C21	80.24 (17)	C5—C6—C21—O1	111.39 (18)
N1—C5—C6—C7	−36.04 (15)	C7—C6—C21—C22	174.40 (14)
C14—C5—C6—C7	93.71 (15)	C5—C6—C21—C22	−67.06 (18)
C20—C5—C6—C7	−153.52 (14)	O1—C21—C22—C23	−13.3 (3)
C21—C6—C7—C8	−68.07 (19)	C6—C21—C22—C23	165.08 (17)
C5—C6—C7—C8	167.58 (14)	C21—C22—C23—C24	−171.84 (17)
C21—C6—C7—C1	167.06 (14)	C22—C23—C24—C29	−168.2 (2)
C5—C6—C7—C1	42.72 (16)	C22—C23—C24—C25	15.0 (3)
N1—C1—C7—C8	−159.55 (14)	C29—C24—C25—C26	0.2 (3)
C2—C1—C7—C8	83.6 (2)	C23—C24—C25—C26	177.1 (2)
N1—C1—C7—C6	−33.95 (16)	C24—C25—C26—C27	1.4 (4)
C2—C1—C7—C6	−150.76 (17)	C25—C26—C27—C28	−1.8 (4)
C6—C7—C8—C13	128.54 (17)	C25—C26—C27—F2	−179.9 (2)
C1—C7—C8—C13	−114.13 (18)	F2—C27—C28—C29	178.6 (2)
C6—C7—C8—C9	−52.7 (2)	C26—C27—C28—C29	0.6 (5)
C1—C7—C8—C9	64.7 (2)	C27—C28—C29—C24	1.1 (4)
C13—C8—C9—C10	−0.2 (3)	C25—C24—C29—C28	−1.4 (4)
C7—C8—C9—C10	−179.01 (17)	C23—C24—C29—C28	−178.5 (2)
C8—C9—C10—C11	0.0 (3)	C14—C5—N1—C4	16.4 (2)
C9—C10—C11—F1	−179.59 (18)	C20—C5—N1—C4	−100.36 (18)
C9—C10—C11—C12	0.4 (3)	C6—C5—N1—C4	142.41 (15)
F1—C11—C12—C13	179.42 (19)	C14—C5—N1—C1	−111.12 (16)
C10—C11—C12—C13	−0.6 (3)	C20—C5—N1—C1	132.15 (15)
C11—C12—C13—C8	0.3 (3)	C6—C5—N1—C1	14.92 (17)
C9—C8—C13—C12	0.0 (3)	C3A—C4—N1—C5	−148.7 (10)
C7—C8—C13—C12	178.86 (17)	C3—C4—N1—C5	−111.4 (3)
N1—C5—C14—C15	61.0 (3)	C3A—C4—N1—C1	−20.5 (10)
C20—C5—C14—C15	−177.7 (2)	C3—C4—N1—C1	16.8 (3)
C6—C5—C14—C15	−59.8 (3)	C2—C1—N1—C5	136.26 (17)
N1—C5—C14—C19	−122.63 (16)	C7—C1—N1—C5	11.79 (18)
C20—C5—C14—C19	−1.30 (18)	C2—C1—N1—C4	2.9 (2)
C6—C5—C14—C19	116.59 (16)	C7—C1—N1—C4	−121.54 (17)
C19—C14—C15—C16	0.3 (3)	O2—C20—N2—C19	−175.54 (17)
C5—C14—C15—C16	176.3 (2)	C5—C20—N2—C19	2.2 (2)
C14—C15—C16—C17	−1.5 (4)	C18—C19—N2—C20	175.4 (2)
C15—C16—C17—C18	1.5 (4)	C14—C19—N2—C20	−3.2 (2)

### Hydrogen-bond geometry (Å, °)

**Table:**

*D*—H···*A*	*D*—H	H···*A*	*D*···*A*	*D*—H···*A*
N2—H2···O2^i^	0.86	2.09	2.922 (2)	164
C9—H9···F2^ii^	0.93	2.55	3.165 (3)	124
C28—H28···Cg1^iii^	0.93	2.97	3.886 (3)	169

Symmetry codes: (i) −*x*+1, −*y*+1, −*z*+1; (ii) −*x*+2, −*y*+1, −*z*+1; (iii) *x*+1, *y*, *z*+1.
